# Needle tract seeding recurrence of pancreatic cancer in the gastric wall with paragastric lymph node metastasis after endoscopic ultrasound-guided fine needle aspiration followed by pancreatectomy: a case report and literature review

**DOI:** 10.1186/s12876-020-1159-x

**Published:** 2020-01-15

**Authors:** Nami Sato, Shigetsugu Takano, Hideyuki Yoshitomi, Katsunori Furukawa, Tsukasa Takayashiki, Satoshi Kuboki, Daisuke Suzuki, Nozomu Sakai, Shingo Kagawa, Takashi Mishima, Eri Nakadai, Rintaro Mikata, Naoya Kato, Masayuki Ohtsuka

**Affiliations:** 1 Department of General Surgery, Graduate School of Medicine, Chiba University, 1-8-1, Inohana, Chuo-ku, Chiba, 260-8677 Japan; 2 Department of Gastroenterology, Graduate School of Medicine, Chiba University, 1-8-1 Inohana, Chuo-ku, Chiba, 260-8677 Japan

**Keywords:** Pancreatic cancer, Endoscopic ultrasound-guided fine needle aspiration, Needle tract seeding, Recurrence, Lymph node metastasis

## Abstract

**Background:**

Endoscopic ultrasound-guided fine needle aspiration (EUS-FNA) has high accuracy and a low complication rate; therefore, it has been widely used as a useful tool for diagnosis of and to determine treatment strategies for pancreatic tumors. Recently, reports of the recurrence of needle tract seeding after EUS-FNA are emerging.

**Case presentation:**

An 83-year-old woman was referred to our hospital to undergo further examination of her pancreatic tumor. Multidetector computed tomography (MDCT) revealed a 25-mm-diameter mass in the pancreatic body. She underwent EUS-FNA (transgastric, 22-G needle, 2 passes) and was subsequently diagnosed with adenocarcinoma. Distal pancreatosplenectomy followed by adjuvant chemotherapy with S-1 for 6 months was performed. The level of carbohydrate antigen 19–9 gradually increased 22 months after surgery, and MDCT, which was performed 3 months later, revealed a 23-mm low-density mass in the stomach and paragastric lymph node swelling. Gastroendoscopy revealed a submucosal tumor, and endoscopic ultrasound revealed a hypoechoic mass in the submucosa of the gastric wall. Partial gastrectomy with lymph node resection was performed. The pathological findings showed adenocarcinoma extending from the subserosa to the submucosa and lymph node metastasis, consistent with a tumor recurrence from the resected pancreatic tumor. She received adjuvant chemotherapy with S-1; recurrence was not observed for 5 months, at the time of this writing.

**Conclusion:**

It is important to pay careful attention to the development of needle tract seeding in patients with pancreatic cancer diagnosed by EUS-FNA. This is the first case of needle tract seeding with lymph node metastasis, highlighting the need for caution and providing novel insight in the postoperative follow-up of patients with pancreatic body/tail cancer.

## Background

Endoscopic ultrasound-guided fine needle aspiration (EUS-FNA) is a minimally invasive sampling technique. Because of its high accuracy [1] and low complication rate [2], EUS-FNA is widely used as a useful tool to diagnose and determine the treatment strategies for pancreatic tumors. However, clinical concerns about peritoneal dissemination or needle tract seeding associated with puncture exist. Although several reports have indicated that preoperative EUS-FNA for pancreatic cancer does not affect postoperative survival or peritoneal recurrence [3, 4], emerging cases of needle tract seeding after EUS-FNA have been recently highlighted. Herein, we report a case of tumor seeding of pancreatic cancer after distal pancreatosplenectomy following EUS-FNA, and we review the literature related to needle tract seeding after EUS-FNA.

## Case presentation

**Fig. 1:**
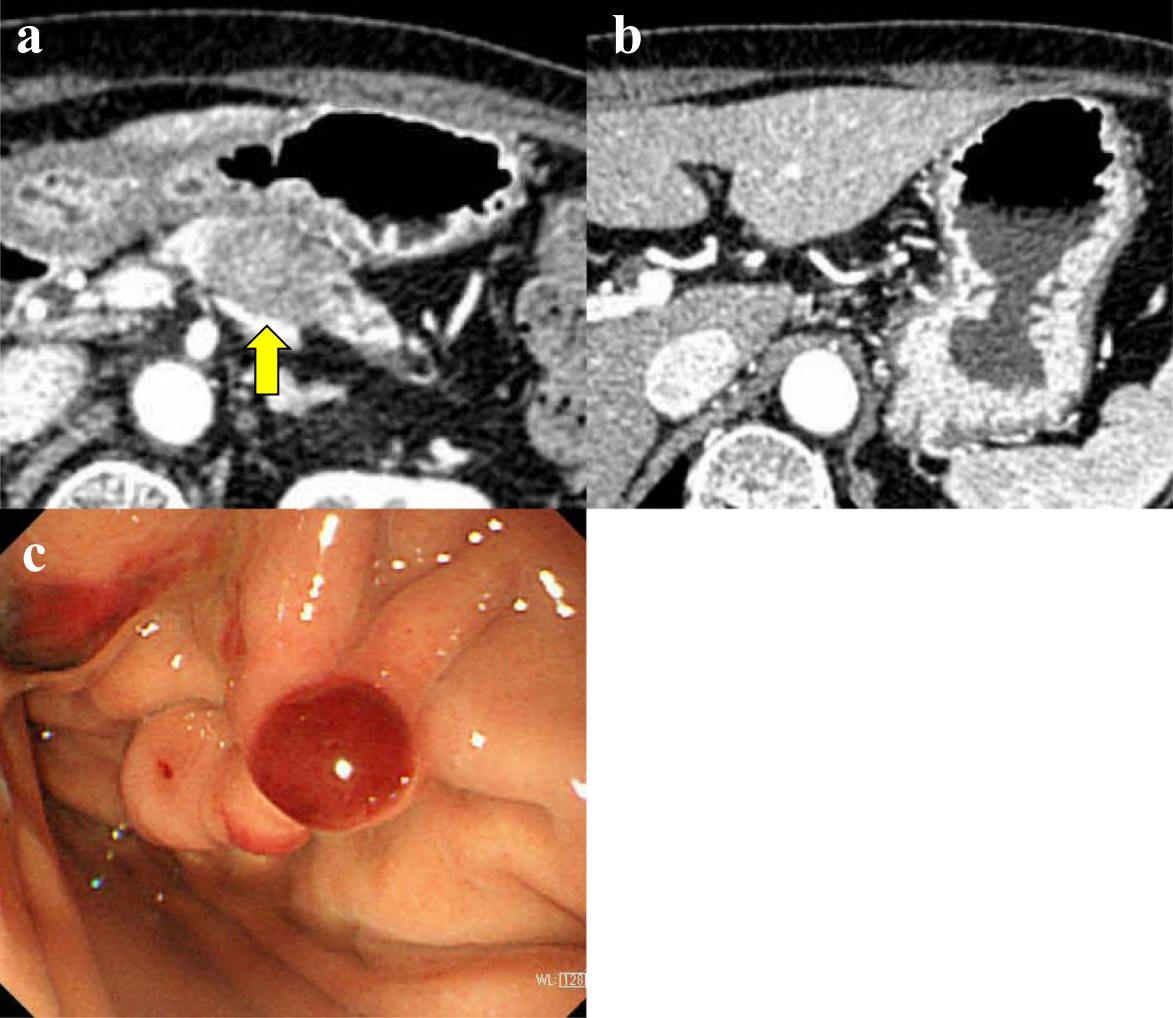
Images prior to the initial operation for pancreatic cancer. **a** Computed tomography revealed a low-density mass in the body of the pancreas (arrow). **b** Lymph node swelling around the stomach before surgery was not observed. **c** The puncture site of fine needle aspiration

An 83-year-old woman with a pancreatic mass identified on abdominal ultrasonography during a medical examination was referred to our hospital. Multidetector computed tomography (MDCT) revealed a 25-mm-diameter low-density mass in the body of the pancreas with dilatation of the main pancreatic duct (Fig. 1a). The carbohydrate antigen 19–9 (CA19–9) level was significantly higher (286 U/mL) than the normal range. To examine the pathological diagnosis, EUS-FNA of the pancreatic tumor was performed using a 22-G needle (SonoTip® 22G, Medicos-Hirata, Tokyo, Japan) passed twice through the gastric wall. During the procedure, early complications were not observed. Subsequently, the patient was diagnosed with adenocarcinoma. Considering that metastatic disease was not observed and her condition was good for her age, she underwent distal pancreatosplenectomy with lymphadenectomy (i.e., anterior radical antegrade modular pancreatosplenectomy). Based on the intraoperative findings, peritoneal dissemination was not observed, and the peritoneal washing cytology was negative for carcinoma cells (CY0). The pancreatic tumor did not invade the gastric wall. The pathological findings resulted in a diagnosis of invasive ductal carcinoma (tub2, Pbt, pTS2 [32 × 25 × 20 mm], infiltrative type, int, INFb, ly3, v2, ne1, pT3, pCH0, pDU0, pS1, pRP1, pPV1sp, pA0, pPL1, pOO0, pN1b [10/20] [#11], pM0, pStage IIB) (according to the 7th edition of the Japanese Pancreas Society classification) with R0 resection (pT2N2M0 pStage III, according to the 8th edition of tumor-node-metastasis (TNM) classification by the American Joint Committee on Cancer/Union for International Cancer Control). According to the pathological examination, there was an invasion on the serosal side of the anterior pancreatic tissue, but not on the stomach. She received adjuvant chemotherapy containing S-1 for 6 months.

**Fig. 2:**
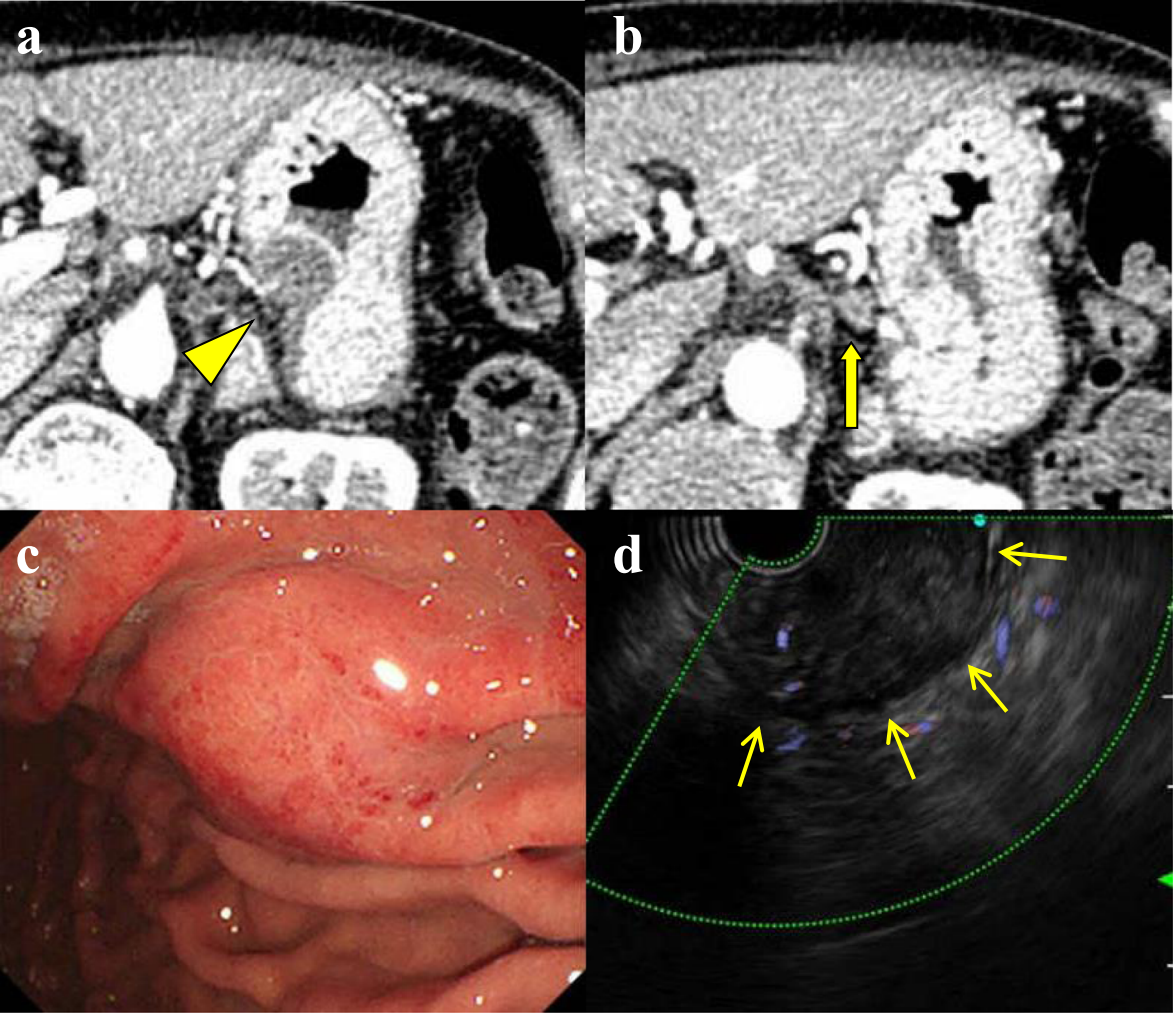
Images at the detection of needle tract seeding in the gastric wall. **a** Twenty-five months after initial pancreatectomy, computed tomography revealed a 23-mm-diameter low-density mass in the wall of the gastric body (arrow head). **b** A lymph node around the stomach was swollen (arrow). **c** Gastroendoscopy revealed an approximately 10-mm-diameter submucosal tumor in the posterior gastric wall. **d** Endoscopic ultrasound revealed a hypoechoic mass in the submucosa (arrows)

**Fig. 3:**
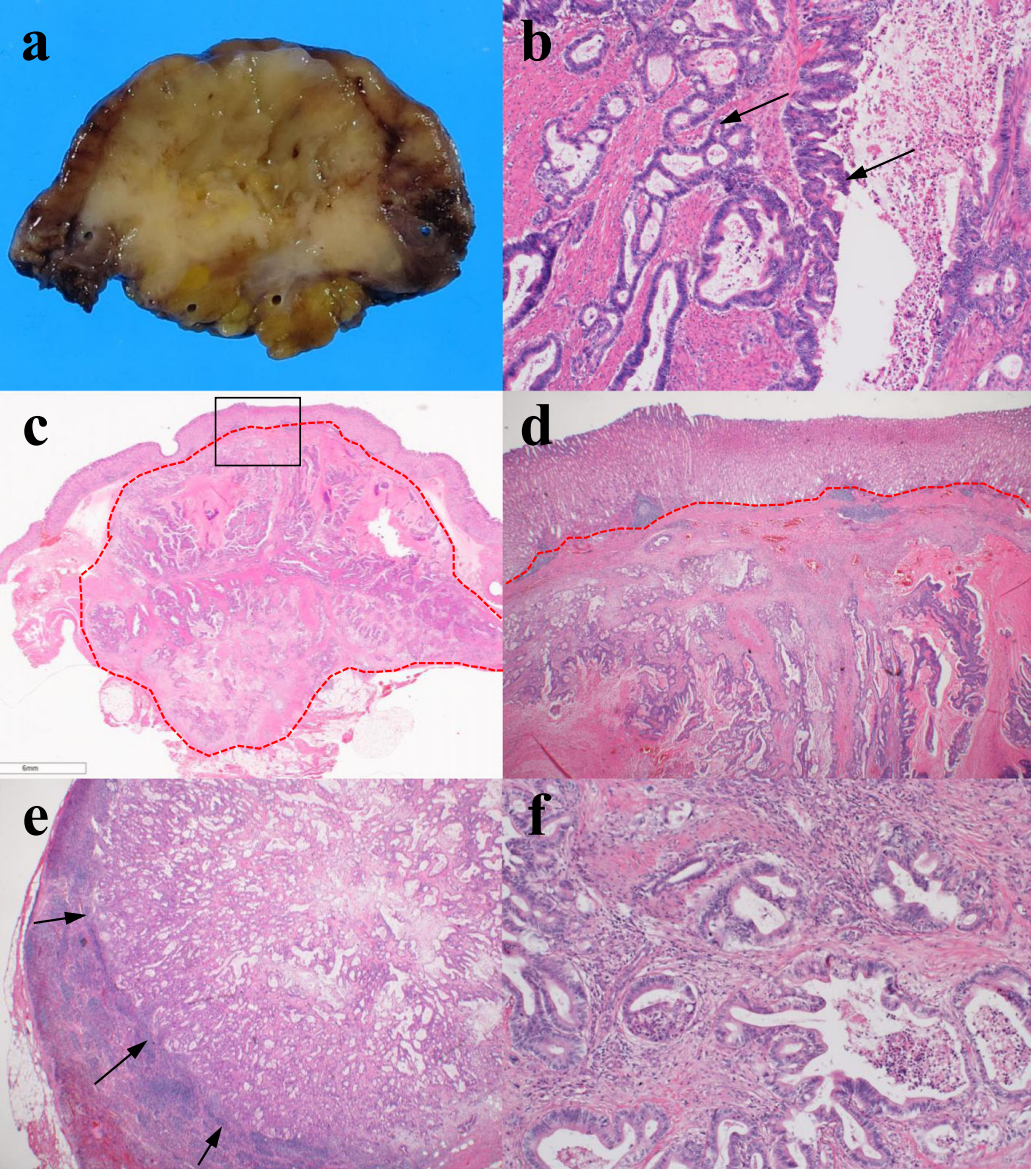
Pathological findings of the resected tumor. **a** Macroscopic image of the resected gastric tumor showed a white hard tumor. **b** Columnar epithelium with atypical nuclei spread in the gastric wall while forming a ductal structure (arrows). These were similar to the histological findings of the previously resected pancreatic cancer. **c** Adenocarcinoma was mainly located from the subserosa to the muscularis propria in the gastric wall, with invasion in the submucosa (within dotted line). **d** An enlargement within the square of (**c**). The mucosal layer was preserved. **e** No. 3 lymph node metastasis was observed in the adenocarcinoma (arrows). **f** Primary pancreatic cancer. From these pathological findings, the submucosal tumor in the gastric wall was diagnosed as arising from needle tract seeding derived from pancreatic cancer

**Fig. 4:**
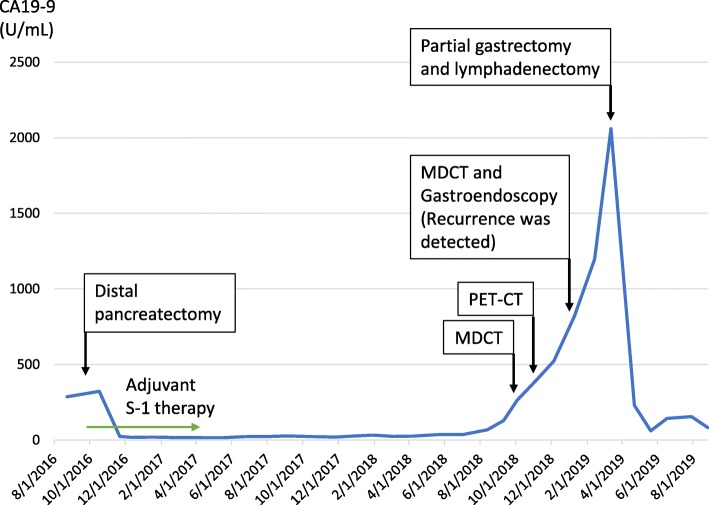
Treatment timeline of the patient

Twenty-two months after surgery, the CA19–9 level increased, but both MDCT and positron emission tomography did not show an evidence of recurrence. Three months later, during a careful follow-up, MDCT revealed a 23-mm low-density mass in the gastric body wall (Fig. 2a) with paragastric lymph node swelling (Fig. 2b). Gastroendoscopy revealed a 10-mm submucosal tumor in the posterior wall of the middle gastric body (Fig. 2c), and endoscopic ultrasound revealed a hypoechoic mass in the submucosa (Fig. 2d). Adenocarcinoma was detected on EUS-FNA, and the gastric mass was diagnosed as a metastasis of pancreatic cancer caused by needle tract seeding. Partial gastrectomy and lymph node resection were performed. Using an electrosurgical knife, all layers of the stomach located with the gastric tumor was resected with a certain margin, and the only hard and swollen lymph nodes were resected. Regarding the intraoperative findings, other metastases or peritoneal disseminations were not observed, and the cytology for ascites was negative. A 25-mm white mass was observed in the resected specimen (Fig. 3a), and histopathological examination indicated adenocarcinoma, extending from the submucosa to the subserosa in the gastric wall (Fig. 3b–d), and paragastric lymph node metastasis (Fig. 3e). She was diagnosed with metastasis of the previous pancreatic cancer derived from needle tract seeding (Fig. 3f). The postoperative course was uneventful, and recurrence was not observed for 5 months, at the time of this writing. The timeline of the treatment for this patient is described in Fig. 4.

## Discussion

In this report, we described a case of pancreatic cancer recurrence in the gastric wall due to needle tract seeding after distal pancreatectomy following EUS-FNA. To the best of our knowledge, this is the first report of a gastric submucosal tumor arising from needle tract seeding along with paragastric lymph node metastasis. Since the first case was reported by Hirooka et al. in 2003, a total of 18 cases including the present case have been described, and 13 cases were reported within the last 5 years (Table 1) [5–21]. Among these cases, there was no pancreatic tumor located in the head of the pancreas. This might be because the EUS-FNA had been performed through the duodenum for pancreatic head lesions, and the site of puncture would have been resected simultaneously with the primary lesion during pancreatoduodenectomy.

**Table 1:** Characteristics of reported needle tract seeding of pancreatic cancer after EUS-FNA

No.	Details of pancreatic cancer		Interval from FNA (months)	Details of needle tract seeding				Outcomes
	Location, Size (mm)	Initial therapy		Symptom	Elevated CA19–9	Size (mm)	Treatment	
1	Pb, 20	DP + Partial Gx	1	No	NA	micro	Partial Gx	Died 25 months after surgery
2	Pt, 8	DP	21	Yes	+	50	ChemoTx	Died 12 months after diagnosis
3	Pt, 28	DP	14	Yes	+	40	Unknown	Unknown
4	Pb, NA	Central pancreatectomy+Adj CRT, gefitinib	36	No	NA	45	Total Gx	Died with metastasis of melanoma
5	Pb, 20	DP	22	NA	–	NA	Unknown	Unknown
6	Pb, 20	DP	8	No	+	12	Partial Gx	Alive 27 months without recurrence after Gx
7	Pb, 25	DP + Adj S-1	28	No	+	32	Subtotal Gx	Unknown
8	Pb, 25	DP	19	No	+	20	Partial Gx	Alive 16 months without recurrence after Gx
9	NA, NA	DP	6	No	NA	NA	Distal Gx+Adj S-1	Re-recurrence at 21 months after Gx
10	Pbt, NA	Radiation therapy	7	No	NA	NA	Unknown	Unknown
11	NA, 30	ChemoTx	3	No	NA	24	ChemoTx	Died 29 months after initial EUS-FNA
12	Pb, 30	DP + Adj S-1	8	No	+	12	Partial Gx+Adj GEM	Alive 18 months without recurrence after Gx
13	Pb, 10	DP + Adj S-1	22	Yes	NA	NA	Partial Gx	Unknown
14	Pt, 37	DP + Adj GEM/S-1	24	No	NA	20	Partial Gx	Unknown
15	Pb, 35	ChemoTx	8	No	NA	NA	DP + Partial Gx	Unknown
16	Pb, 15	DP + Partial Gx	1	No	+	micro	Partial Gx	Died 18 months after surgery
17	Pb, 34	DP + Partial Gx	4	No	NA	micro	Partial Gx	Alive 18 months after surgery
Our case	Pb, 32	DP + Adj S-1	25	No	+	25	Partial Gx + lymph node	Alive 5 months without recurrence after Gx

*EUS-FNA* Endoscopic-ultrasound-guided fine needle aspiration, *CA19–9* carbohydrate antigen 19–9, *Pb* Pancreatic body, *Pt* Pancreatic tail, *Pbt* Pancreatic body and tail, *NA* Not applicable, *DP* Distal pancreatectomy, *Adj* Adjuvant, *ChemoTx* Chemotherapy, *CRT* Chemoradiation therapy, *GEM* Gemcitabine, *Gx* Gastrectomy

The optimal treatment and long-term prognosis of needle tract seeding are still unknown. In general, it is rare to resect recurrent lesions in patients with postoperative pancreatic cancer since the median survival time from the detection of recurrence to death is 3–10 months [22, 23]. However, a recent systematic review, which analyzed the data of 301 postoperative patients with isolated recurrence of pancreatic cancer, showed that the median overall survival was 26.0 months (range, 0–112 months), and median disease-free survival was 14.2 months (range, 4–29 months) after the resection of recurrence sites [24]. Although these findings were analyzed in a heterogeneous and limited number of patients, these data showed that some patients may benefit from surgery. This suggests that surgery may contribute to the improvement in the prognosis in the case of gastric wall recurrence due to needle tract seeding considering that there are no other recurrent lesions. To improve the outcomes of patients with needle tract seeding by surgical procedures, early detection of recurrence is significantly crucial. Based on a review of the literature, subjective symptoms are clinically insignificant in establishing the diagnosis because 14 of the 17 patients (82.4%) were asymptomatic at the time of recurrence detection. In contrary, increased CA19–9 levels were observed in 8 of the 9 cases (88.9%). Therefore, CA19–9 might be useful for the early detection of recurrence due to needle tract seeding during the postoperative follow-up of patients.

Although several reports have suggested that the translocation of malignant cells is associated with EUS-FNA [18, 25], the developmental process of needle tract seeding is unclear. Because the number of reports is significantly small, it remains unknown whether tumor factors or fine needle aspiration procedure factors such as needle size, the number of puncture sites, and the number of needle passes are significantly associated on the occurrence of needle tract seeding. In addition, needle tract seeding of pancreatic cancer will be considered a significantly important problem in the future. A randomized controlled trial that compared neoadjuvant chemotherapy using gemcitabine and S-1 with upfront surgery (Prep-02/JSAP05) demonstrated the significant survival benefits of neoadjuvant chemotherapy for patients with resectable pancreatic cancer [26]. Considering this result, neoadjuvant chemotherapy can be considered a potentially beneficial treatment for pancreatic cancer, and simultaneously, the pathological diagnosis obtained by EUS-FNA is essential before performing an initial treatment. Hence, a prospective cohort study comprising a large sample size is required to confirm the detailed clinical characteristics of needle tract seeding.

In conclusion, patients with pancreatic body or tail cancer diagnosed by EUS-FNA should pay careful attention on the occurrence of needle tract seeding and lymph node metastasis. Further cumulative cases are required to elucidate the accurate frequency, optimal treatment, and long-term outcomes.

## Data Availability

The datasets used and/or analyzed during the current study are available from the corresponding author on a reasonable request.

## Abbreviations

CA19–9: Carbohydrate antigen 19–9
EUS-FNA: Endoscopic-ultrasound-guided fine needle aspiration
MDCT: Multidetector computed tomography
